# Urban runoff treatment using nano-sized iron oxide coated sand with and without magnetic field applying

**DOI:** 10.1186/2052-336X-11-43

**Published:** 2013-12-20

**Authors:** Mehdi Khiadani (Hajian), Mansur Zarrabi, Maryam Foroughi

**Affiliations:** 1 School of Engineering, Edith Cowan University, WA, Australia; 2 Department of Environmental Health Engineering, Environment Research Center, School of Health, Isfahan University of Medical Sciences, Isfahan, Iran; 3 Department of Environmental Health Engineering, Faculty of Health, Alborz University of Medical Sciences, Karaj, Iran; 4 Department of Environmental Health Engineering, School of Health, Hamedan University of Medical Sciences, Hamedan, Iran

**Keywords:** Urban runoff, Iron oxide nano particles, Sand filter, Magnetic field

## Abstract

Increase of impervious surfaces in urban area followed with increases in runoff volume and peak flow, leads to increase in urban storm water pollution. The polluted runoff has many adverse impacts on human life and environment. For that reason, the aim of this study was to investigate the efficiency of nano iron oxide coated sand with and without magnetic field in treatment of urban runoff. In present work, synthetic urban runoff was treated in continuous separate columns system which was filled with nano iron oxide coated sand with and without magnetic field. Several experimental parameters such as heavy metals, turbidity, pH, nitrate and phosphate were controlled for investigate of system efficiency. The prepared column materials were characterized with Scanning Electron Microscopy (SEM) and Energy Dispersive X-ray analysis (EDXA) instruments. SEM and EDXA analyses proved that the sand has been coated with nano iron oxide (Fe_3_O_4_) successfully. The results of SEM and EDXA instruments well demonstrate the formation of nano iron oxide (Fe_3_O_4_) on sand particle. Removal efficiency without magnetic field for turbidity; Pb, Zn, Cd and PO_4_ were observed to be 90.8%, 73.3%, 75.8%, 85.6% and 67.5%, respectively. When magnetic field was applied, the removal efficiency for turbidity, Pb, Zn, Cd and PO_4_ was increased to 95.7%, 89.5%, 79.9%, 91.5% and 75.6% respectively. In addition, it was observed that coated sand and magnetic field was not able to remove NO_3_ ions. Statistical analyses of data indicated that there was a significant difference between removals of pollutants in two tested columns. Results of this study well demonstrate the efficiency of nanosized iron oxide-coated sand in treatment of urban runoff quality; upon 75% of pollutants could be removed. In addition, in the case of magnetic field system efficiency can be improved significantly.

## Introduction

Storm water runoff from paved surfaces can carry large loads of various pollutants including heavy metals, hydrocarbons, nutrients and pathogens [1-3]. These pollutants may originate from motorized vehicle emissions, automobile tires, brake pads, corrosion of pavement, chemical deposition on or near the pavement surface and anthropogenic activities [1,4]. On the other hand, since impervious surfaces such as roofs and roads dominate the land cover of urbanized areas, cities suffer from increased and more intense runoff, reduced groundwater recharge and runoff water quality [5], increased peak flows [6] and hydrological, physico-chemical and consequent biological disturbance of the receiving waters [7,8]. Special attention should be paid to heavy metals in storm water runoff due to their toxicity [9]. Some of the most frequently reported metals in storm water are cadmium (Cd), lead (Pb) and zinc (Zn) that are considered to be of the great concern. Concentrations of these ions in storm water commonly exceed surface water quality guidelines by 10 times or more [10]. Release of heavy metals into natural receiving waters can cause accumulation of non-biodegradable metals in the environment, causing both short-term and long-term adverse effects on human life [11]. Phosphorus (P) is the most commonly present substance in freshwater bodies subject to eutrophication [10]. Excess Nitration (N) in storm water leads to saturation of nitrogen, water bloom and associated water-quality problems [12].

Several methods have been developed and used for treatment of storm water runoff from urban area. Natural and constructed wetlands, for example, have been investigated as practical alternatives for treating runoffs in several studies. These systems allow reducing primarily particulate pollutants. The constructed wetlands and retention ponds require large area and these systems were mostly applied for pollution source areas at catchment scale. Filtration of storm water through a filter system filled with adsorbents (e.g. zeolite, peat, granular activated carbon or sand) is another possible treatment method that is relatively recent innovation for treatment of runoff [13].

Iron (hydr) oxide-coated sand (IOCS) has shown to have high efficiency in removing microorganisms, turbidity and heavy metals [14]. Many studies have used IOCS to remove lead [15], arsenic [16], nickel and copper [17], organic matter [18], humic [19,20], and phosphate [21] from aqueous solution and/or wastewater. On the other hand, magnetic treatment of wastewater can be applied to eliminate heavy metals, color, phosphates and oil at low concentration. Some studies have reported that magnetic field affects properties of water such as light absorbance, pH, zeta potential and surface tension. However, these idea have not always been confirmed [22]. In recent years, attention has been paid to the possibility of enhancing treatment of wastewater by static magnetic field. However information is not available about the effect of magnetic field on the biological degradation of wastewater organic matter [23]. Magnetic field was used to improve anaerobic ammonium oxidation in which nitrogen removal increased by 30% with 25% less time [24]. In addition, magnetic field has been used for formaldehyde biological degradation with 30% increases in removal efficiency if compared with other technologies [25].

For that reason, the aim of this study was to investigate the efficiency of sand filter coated with nano iron oxide for treatment of urban runoff. In addition to this, magnetic field was applied to the coated sand filter to find out if the magnetic field can further improve the treatment of urban runoff.

## Materials and methods

### Chemicals and reagents

All chemical materials used in this study were provided from Merck Company. Synthesized urban runoff containing lead, zinc, cadmium, nitrate and phosphate, were prepared using stock solution of PbCl_2_, ZnSO_4_.7H_2_O, (CH_3_COO) 2Cd.2H_2_O, KNO_3_ and K_2_HPO_4_, respectively. Turbidity and pH of synthesized runoff were adjusted using Kaolin and, 1 N HNO_3_ and NaOH solutions, respectively. Samples were collected in 100 mL bottles. The characteristics of the synthesized runoff are presented in Table 1.

**Table 1:** **Characteristics of synthesized runoff**[26]

**Pollutant**	**Concentration**
Turbidity(NTU)	60
Lead(mg/l)	2.37
Zinc(mg/l)	2.54
Cadmium(mg/l)	0.52
Nitrate(mg/l)	4-5
Phosphate(mg/l)	9-10
pH	6.5-7.5

### Sand filter media

Filter media was packed with local quarry sands ranging between 0.85 and 2.36 mm. Before used in column and coating, The sand was soaked in 8% nitric acid solution overnight, rinsed with deionised water to pH = 7.0 and dried at 105°C. The sands were coated with iron oxide according the method suggested by Mostafa et al. [27]. The solution of Fe(III) was prepared by dissolving reagent grade FeCl_3_·6H_2_O in deionized water. The solution was stirred with a magnetic stirrer at 200 rpm and 0.5 M NaOH solution was added for adjusting pH at 9.5 ± 0.1 and mixed for 5 min. The mixed solution was introduced to 100 g sand in a conical flask and was placed in a temperature-controlled shaker at 60 ± 1°C, then stirred at 200 rpm for 24 h. After that, the coated sand was dried in an oven at 105 ± 1°C for 24 h. Finally, the prepared sands particle was washed 5–7 times with deionized water for remove uncoated iron particle, dried at 60 ± 1°C for 24 h and used for future experiment.

### Column tests

**Figure 1:**
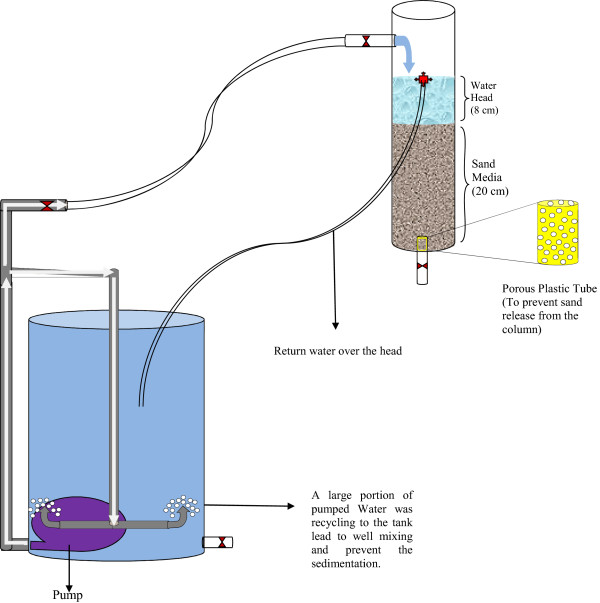
**Schematic of the pilot**-**scale system used in this study.**

Columns used in this study were made of Plexi Glass with 5 cm internal diameters, 35 cm height, and 20 cm medium bed depth. Since the columns were operated in down flow mode, to avoid flow channelization, an additional 8 cm water head was provided on the columns surface. Two magnets of 20 cm high with 0.7 T (Tesla) magnetic charge density was mounted around one of the columns to investigated the effects of magnetic field on the removal efficiency of the pollutants from the synthesized runoff. The flow rate of runoff from both columns was adjusted at 20 mL/min leading to 20 min retention time [14] of runoff in the column. Schematic of the present pilot is shown in Figure 1 (Magnets are not shown). The samples were obtained from the bottom of columns and then were analyzed for investigated parameters. Each column was operated for 60 hours, during which 10 samples were collected. The experimental data were conducted in triplicate and average value was considered. Before analysis of nitrate and heavy metals, pH of the samples was reduced to 2 by adding sulfuric and nitric acids, respectively [28].

### Instruments

Concentration of lead (Pb), zinc (Zn) and cadmium (Cd) in effluent were determined using a Perkin Elmer 2380 atomic absorption spectrometer. Nitrate and phosphate was determined using UV–vis spectrophotometer (HACH DR5000). Turbidimeter (Euteoh Instruments TN 100) was used to measure turbidity. Schott pH meter model CG-824 was used for pH analysis. Size and characteristics of nano particles was determined using Scanning Electron Microscope (SEM) and Energy Dispersive X-ray Analysis (EDXA).

## Results and discussion

### Adsorbent characterization

**Figure 2:**
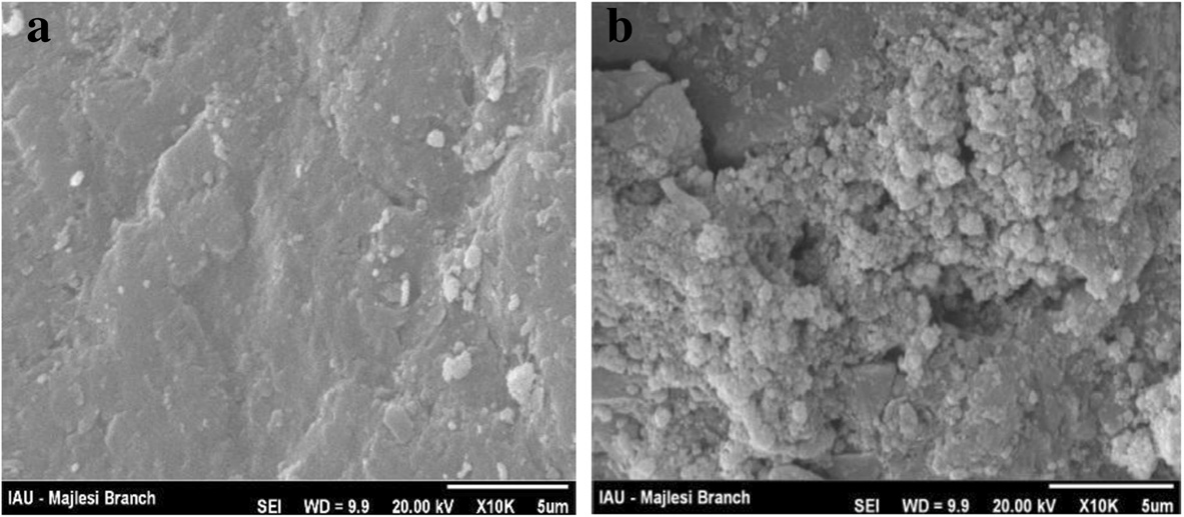
**Scanning electron micrographs of natural sand (a), and coated sand with nano iron oxide (b).**

Figure 2a shows that the surface of uncoated sand is relatively smooth; however, the surface of coated sand with iron oxide nano particles shown in Figure 2b is rough. Due to deposition of iron oxide particles on the sand surface, the coated sand has more microspores and its specific surface area may be high as in comparison with uncoated sand. This is in agreement with the results of Lai et al. who reported 0.85 m^2^ /g and 2.76 m^2^ /g for natural and coated sand, respectively [29].

**Figure 3:**
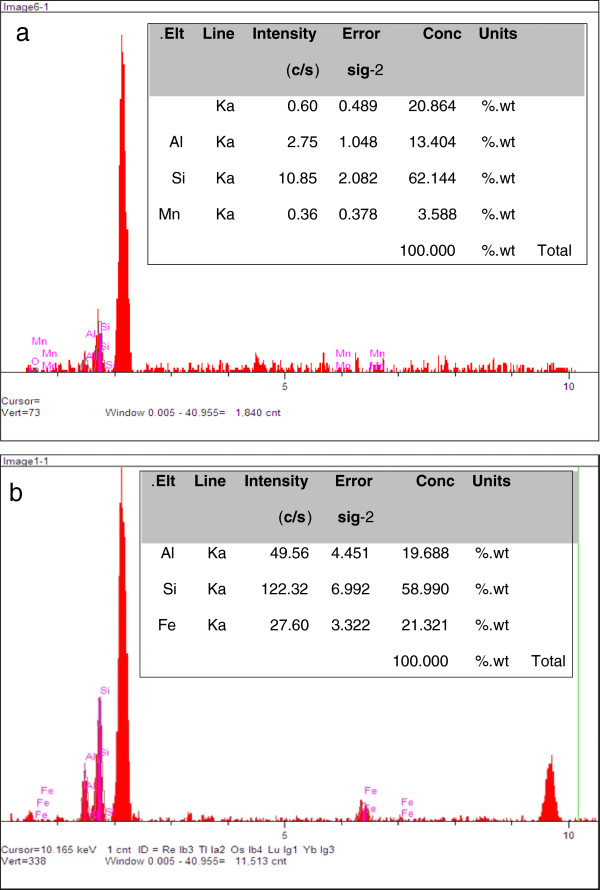
**Energy dispersive X-ray analysis spectra of natural sand (a), and coated sand with nano iron oxide (b).**

Elemental composition of the sand was determined using EDXA spectra analysis. EDXA analysis of the unmodified sand showed the main components of the sand were Si (62.1%) and O (20.8%) (Figure 3a). After coating of sand with nano iron oxide, the iron content of the sand was increased to 21% of its elemental composition (Fig 3b). This is in agreement with result of Hsu et al. [15]; showing the iron content of the sand was increased by 6% after coating with nano iron oxide. In contrast to our study, the size of coated particle in Hsu and co-worker study was not in nano size. Coating with nano particles caused the iron oxide penetrate to deeper layers of the sand and provide a larger interlayer space which may lead to larger micro pore diameters and spaces [15].

### Removal of turbidity

**Figure 4:**
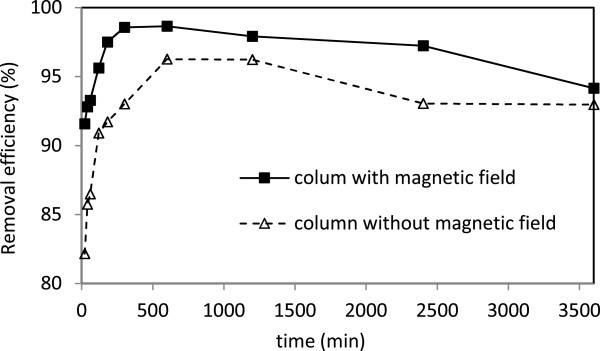
The removal efficiency of turbidity in columns with and without magnetic field.

Average removal efficiency of turbidity from column without magnetic field was achieved 90.8% (Figure 4). The removal mechanisms in this column were filtration, settling and adsorption [30]. Once the magnetic field was applied to the column, the removal efficiency increased to 95.7%. In fact, colloidal stability is influenced by the application of magnetic field, possibly indicating a reduction in charge density within the stern layer. Also it has been proposed that magnetic field reduces zeta potential of colloidal particles causing particle instability, aggregation and a more rapid sedimentation [22].

### Removal of lead, zinc and cadmium

**Figure 5:**
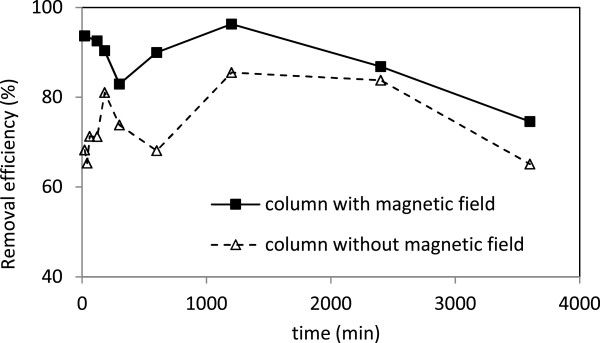
The removal efficiency of lead in columns with and without magnetic field.

**Figure 6:**
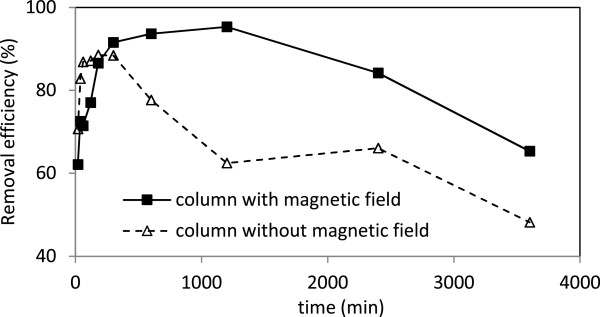
The removal efficiency of zinc in columns with and without magnetic field.

**Figure 7:**
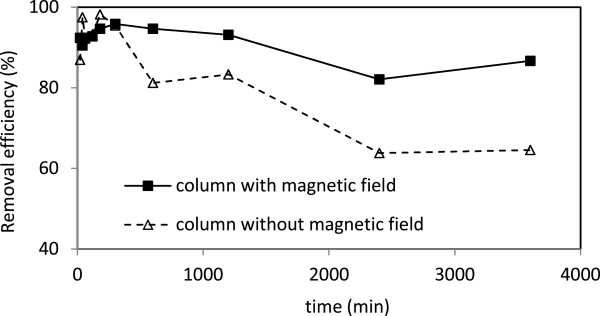
The removal efficiency of cadmium in columns with and without magnetic field.

Coating sand with nano iron oxide without magnetic field improved the removal efficiency of lead, zinc and cadmium to 73.3%, 75.8% and 86.1%, respectively (Figures 5, 6 and 7). The 54% removal efficiency of lead from the sand filter without coating has been reported [31]. X-ray photoelectron spectroscopy (XPS) surface analysis of Pb(II)-adsorbed on Mg/Al layered double hydroxide (LDH) surfaces revealed that the preferred reaction between LDH and Pb(II) are surface adsorption and precipitation [15]. Thus treatment with metal oxides increases the number of adsorption sites significantly. In case of zinc and cadmium, it seems that due to the existence of agent groups at the surface, they lead to adsorption of the cations [32].

With applying the magnetic field to the coated sand, the removal efficiency of lead, zinc and cadmium was increased to 89.6%, 80%, and 91.5%, respectively; which are in agreement with previously published work [22]. There are not much information about the mechanism of magnetic field on soluble ions, however, magnetic force leads to increase in electrostatic interaction between positively charged ions such as lead, zinc and cadmium with adsorbent surface; followed with increase in metals adsorption capacity. It may be due to releases of free electron from adsorbent surface to bounding with metals ions. Another reason may be due to decreases in zeta potential [22] leading to increase in metal bounding with present medium.

Achak et al. reported that solution conductivity of the effluent from sand filter was reduced. They declared that this reduction is due to the adsorption of the cations on the colloids negatively charged [33]. On the other hand, it has been reported that magnetic field reduce zeta potential [22], which causes colloids instability and increases agglomeration and then speeding up settling. Moreover, adsorption to suspended solids occurs before settling and filtration or adsorption to substrate [30]. Therefore, an increase in removal efficiency may be is due to the occurrence of two subsequent phenomenon’s: adsorption of the cations on the colloids pollutants in the first stage, and enhanced the colloids agglomeration and settling, because of zeta potential reduction, in the second stage.

### Removal of phosphate

**Figure 8:**
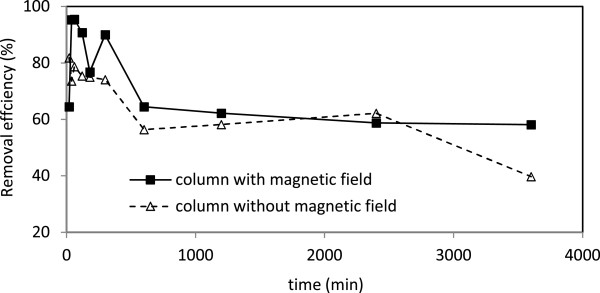
The removal efficiency of phosphate in columns with and without magnetic field.

As indicated in Figure 8, 69.1% phosphate in the column without magnetic field was removed. Our results were in agreement with previously published work for removal of phosphate with sand filter by adsorption [21,33]. In contrast, Hatt et al. reported that the sand filter is not a suitable treatment option for removal of phosphate [31].

SEM photographs in this study showed that iron oxide are well deposited on the sand, resulting in increase of the surface sites and so chance of phosphate ions adsorption. Scholes et al. reported that adsorption is physico-chemical adherence that is controlled by factors such as the particulate surface area and surface composition [30]. Moreover Achak et al. emphasized that ions are well retained by the cations of the sand such as iron and aluminum oxides [33]. Therefore, in addition to increase of surface area, the presence of iron on the surface caused to promote of adsorption, due to adsorption of the ions onto iron deposits.

Since phosphate and arsenic are in the same group in the periodic table and their ionic charge and size are very similar [27], Boujelben et al. declared phosphate ions are as models for removal of similar pollutant (i.e. arsenates and antimonies) [21]. Besides, it has been reported that one reason for higher efficiency of iron oxide coated sand for As (III) may be is due to formation of ferric hydroxide in the aqueous solution responsible for the co-precipitation of the ions on the surface of the adsorbent [34]. Therefore, it well demonstrated that only adsorption is not contributed in the phosphate removal. According this, Mostafa et al. declared that if desorption rate was slower than adsorption rate, chemical mechanism is contribute in the ion removal, and since this relationship was established in their study, they conclude that chemical bonding was formed between arsenic ions and the coated surface [27]. Therefore, in addition to adsorption, formation of chemical bonding between phosphate ions and the coated surface was contributed in the phosphate ion removal. The coated sand with nano iron oxide with magnetic field increased the removal efficiency of phosphate to 75%. As it was explained previously, magnetic field could reduce zeta potential and causes insatiability which leads to adsorption and agglomeration of phosphate. Moreover, magnetic force breaks hydrogen bonds between water molecules, so the ions become separated and combine with elements (such as Pb, P, Ni, *etc*.) and precipitate [22].

### Removal of nitrate

**Figure 9:**
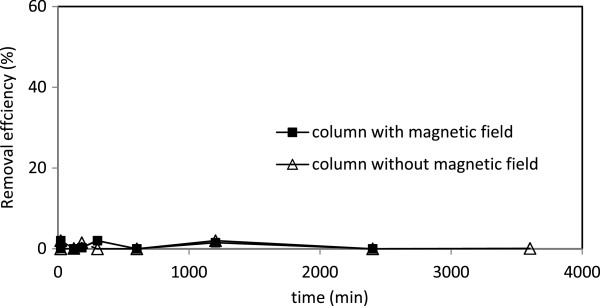
The removal efficiency of nitrate in columns with and without magnetic field.

Neither the sand filter coated with nano iron oxide nor magnetic field was able to remove nitrate form the synthesized runoff (Figure 9). It has been reported that biochemical process may help to remove nitrate [31]. This is basically due to the formation of biofilm which allows oxidation of all nitrogen forms in the filter [33].

Statistical analyses of data indicated that there was a significant difference between removals of pollutants in two columns. In case of turbidity, lead, zinc, cadmium and phosphate, prob > |*t*| is 0.005, 0.03, 0.03, 0.005, and 0.007 respectively (all less than 0.05).

## Conclusions

In present study, natural sand and nano sized iron coated sand with and without applying magnetic field was used for treatment of synthetic urban runoff. Results indicate that nano sized iron oxide-coated sand has a significant efficiency to improve urban runoff quality. Nano iron coated sand was able to remove 75% of pollutant in average. In addition, in the case of magnetic field, removal efficiency was improved significantly; showing effectives of nano iron coated sand in the presence of magnetic field. Our results well demonstrate that present system is effective methods as compared with other existing methods for treatment of urban runoff containing, phosphate, turbidity, cadmium, lead and zinc. Although the real urban runoff must be applied to achieve real results and further studies are needed to implement of this system on a large scale.

## Competing interests

The authors declare that they have no competing interests.

## Authors’ contributions

MK (Hajian) was involved in experimental parts and takes the initial samples from pilot plant. MZ reviewed the final manuscript and also involved in experimental parts. MF was involved in the design of the Study, analysis and interpretation of data, drafting the initial manuscript. All authors read and approved the final manuscript.
